# Refining wet lab experiments with *in silico* searches: A rational quest for diagnostic peptides in visceral leishmaniasis

**DOI:** 10.1371/journal.pntd.0007353

**Published:** 2019-05-06

**Authors:** Bruno Cesar Bremer Hinckel, Tegwen Marlais, Stephanie Airs, Tapan Bhattacharyya, Hideo Imamura, Jean-Claude Dujardin, Sayda El-Safi, Om Prakash Singh, Shyam Sundar, Andrew Keith Falconar, Bjorn Andersson, Sergey Litvinov, Michael A. Miles, Pascal Mertens

**Affiliations:** 1 Coris BioConcept, Gembloux, Belgium; 2 Department of Biomedical Sciences, University of Antwerp, Antwerp, Belgium; 3 Faculty of Infectious and Tropical Diseases, London School of Hygiene and Tropical Medicine, London, United Kingdom; 4 Faculty of Medicine, University of Khartoum, Khartoum, Sudan; 5 Department of Medicine, Institute of Medical Sciences, Banaras Hindu University, Varanasi, Uttar Pradesh, India; 6 Departamento de Medicina, Universidad del Norte, Barranquilla, Colombia; 7 Department of Cell- and Molecular Biology, Karolinska Institutet, Stockholm, Sweden; 8 Aptum Biologics Ltd., Southampton, United Kingdom; Christian Medical College, Vellore, INDIA

## Abstract

**Background:**

The search for diagnostic biomarkers has been profiting from a growing number of high quality sequenced genomes and freely available bioinformatic tools. These can be combined with wet lab experiments for a rational search. Improved, point-of-care diagnostic tests for visceral leishmaniasis (VL), early case detection and surveillance are required. Previous investigations demonstrated the potential of IgG1 as a biomarker for monitoring clinical status in rapid diagnostic tests (RDTs), although using a crude lysate antigen (CLA) as capturing antigen. Replacing the CLA by specific antigens would lead to more robust RDTs.

**Methodology:**

Immunoblots revealed *L. donovani* protein bands detected by IgG1 from VL patients. Upon confident identification of these antigens by mass spectrometry (MS), we searched for evidence of constitutive protein expression and presence of antigenic domains or high accessibility to B-cells. Selected candidates had their linear epitopes mapped with *in silico* algorithms. Multiple high-scoring predicted epitopes from the shortlisted proteins were screened in peptide arrays. The most promising candidate was tested in RDT prototypes using VL and nonendemic healthy control (NEHC) patient sera.

**Results:**

Over 90% of the proteins identified from the immunoblots did not satisfy the selection criteria and were excluded from the downstream epitope mapping. Screening of predicted epitope peptides from the shortlisted proteins identified the most reactive, for which the sensitivity for IgG1 was 84% (95% CI 60—97%) with Sudanese VL sera on RDT prototypes. None of the sera from NEHCs were positive.

**Conclusion:**

We employed *in silico* searches to reduce drastically the output of wet lab experiments, focusing on promising candidates containing selected protein features. By predicting epitopes *in silico* we screened a large number of peptides using arrays, identifying the most promising one, for which IgG1 sensitivity and specificity, with limited sample size, supported this proof of concept strategy for diagnostics discovery, which can be applied to the development of more robust IgG1 RDTs for monitoring clinical status in VL.

## Author summary

Visceral leishmaniasis (VL) is a neglected tropical disease caused by protozoan parasites of the *Leishmania donovani* complex. Without treatment, VL is fatal. Although diagnostic techniques, mainly based on the detection of anti-*Leishmania* antibodies are available, invasive procedures such as microscopy from spleen or bone marrow aspirates are still required for the diagnosis of seronegative VL suspects, for the detection of recurrent cases and to confirm cure after successful treatment. Previous investigations showed the potential of IgG1 as a biomarker of post-chemotherapeutic relapse for VL in rapid diagnostic tests (RDTs) sensitised with crude lysate antigen (CLA). Here we employed *in silico* tools to search for desired protein features in a large number of *L. donovani* antigens detected by human IgG1 in western blots. We then employed prediction algorithms to profile epitopes from the shortlisted proteins. We screened a panel of high-scoring peptides in a high-throughput manner using arrays, with low reagent consumption. The most reactive peptide was adapted to RDTs, showing promising results of both sensitivity and specificity. This peptide has the potential of replacing the CLAs in IgG1 RDTs. Thus we believe that *in silico* tools can be used to optimise wet lab experiments for a rational search of biomarkers.

## Introduction

The leishmaniases comprise a group of vector-borne diseases caused by parasites of the genus *Leishmania*. The visceral form—visceral leishmaniasis (VL), also known as kala-azar (Hindi for ‘black fever’) affects internal organs such as liver, spleen and bone marrow, and leads to death if left untreated. VL is caused by parasites of the *Leishmania donovani* complex and in 2015 over 23.000 new cases were reported to the world health organization (WHO) worldwide [1] while an overall case-fatality rate of 10% has been estimated [2]. VL is diagnosed by a combination of clinical symptoms, including prolonged fever, weight loss, hepatosplenomegaly and malaise, and the detection of parasite-specific immunoglobulins (Igs). The recombinant protein rK39, a fragment of a *Leishmania infantum* kinesin-like gene, was described in 1993 [3] and remains the most widely used antigen for VL serodiagnosis. Nonetheless, novel and improved antigens are still required to complement the use of the rK39, to improve sensitivity in eastern Africa [4, 5] and to determine cure after successful chemotherapy (versus relapse).

The recombinant chimeric protein rK28, which is a derivative of the rK39, incorporating the first two repeats of a Sudanese *L. donovani* kinesin flanked by proteins HASPB1 and HASPB2, was engineered to address low sensitivity values reported from eastern Africa. Although a slight better performance compared to the rK39, both in terms of sensitivity and specificity, was reported with Sudanese VL patients [6], important variations in sensitivity amongst different rK39 rapid diagnostic test (RDT) manufacturers were also reported from that region [7]. Moreover, false positive rates as high as 19.5% in eastern African patients were described [8], meaning that further investigations are still required to confirm the relevance of the rK28 as well as its superiority over the rK39-based diagnostic tests for VL in eastern African patients. Due to Igs persistence even after complete parasite clearance [9–13], neither the rK39 nor the rK28 commercial diagnostic kits can be employed to determine cure after successful chemotherapy.

There has been a dramatic reduction in genome sequencing costs, accompanied by an exponential increase in the number of available sequences in public repositories [14, 15]. The first *Leishmania* spp. genome sequencing was completed in 2005 [16]. More recently, the advent of high-throughput technologies has enabled the completion of the whole genome sequencing of *L. donovani*, the causative agent of VL in the Indian subcontinent [17]. Moreover, the availability of next-generation sequencing made it possible to perform whole transcriptome sequencing. RNA sequencing (RNA-seq) generates data on the transcriptome at a specific stage of a pathogen life cycle or in a specific culture condition of an organism. The growing number of available, high quality, whole genome sequences has become a central element in the area of comparative genomics, which has contributed greatly to a better understanding of multiple aspects of the leishmaniases, including determinants of disease phenotype [18], mode of action of drugs [19] and parasite biology [20].

Igs are a major component of the immune system. They bind specific regions of pathogens’ proteins (epitopes), tagging them for clearance by the immune system. Epitopes can be divided into linear (a continuous stretch of amino acids (AAs)) and discontinuous (where non-proximal residues are brought together by protein folding) and can be identified by functional and structural studies (e.g. X-ray crystallography), while *in silico* epitope prediction algorithms are gaining popularity. The early prediction methods of linear B-cell epitopes were mainly based on propensity scales [21]. More recently, machine learning methods have been employed to improve prediction performance [22–24]. The prediction of discontinuous epitopes still depends on the availability of 3D structures. *In silico* tools can also be used to locate antigenic domains from DNA sequences or peptide sequences influencing protein localisation within cells.

Diagnostic research can incorporate genomics, transcriptomics, proteomics as well as bioinformatic prediction algorithms for the discovery of new biomarkers. Such a systematic ‘omics’ approach has been applied alone for the discovery of vaccine candidates [25–27] as well as for diagnostic biomarkers [28]. These *in silico* searches can also be applied downstream of wet lab experiments, refining their output for a rationalised search.

Mass spectrometry (MS) can be used to identify proteins from wet lab experiments (e.g. immunoblots). Comparative genomics enables identification of species-specific genes, while levels of life-stage specific proteins can be estimated using publicly available RNA-seq data. *In silico* prediction algorithms can be employed to infer protein localisation, search for antigenic domains as well as to predict linear B-cell epitopes. Synthetic peptides can then be incorporated into arrays, enabling the screening of a large number of candidates in pilot serological assays. Promising candidates can be adapted and tested in RDTs, a format suitable for field use.

Here we employed comparative genomics as well as *in silico* algorithms to reduce drastically the excessive number of *L. donovani* protein candidates recognised by human IgG1, which has been shown to be a potential indicator of post-chemotherapeutic relapse in VL, using RDT prototypes sensitised with a *L. donovani* crude lysate antigen (CLA) [29]. By screening a large number of predicted epitopes from selected candidate antigens containing desired protein features, we identified one peptide specifically recognised by IgG1 in arrays. This peptide was also tested in prototype RDTs with VL patient sera as well as sera from nonendemic healthy controls (NEHCs), showing promising results of both sensitivity and specificity on a small sample size. Thus we propose that this approach is a valid proof of concept for the discovery of diagnostic peptides, which can be used to replace the CLA in IgG1 RDTs, leading to the production of cheaper and more robust RDTs for monitoring clinical status in VL.

## Methods

### Ethics statement

In India, the collection of samples was approved by the Ethics Committee of Banaras Hindu University, Varanasi while in Sudan the approval for collection and research was granted by the Ethical Research Committee, Faculty of Medicine, University of Khartoum and the National Health Research Ethics Committee, Federal Ministry of Health, Sudan. Written informed consent was obtained from all adult subjects included in the study or from the parents or guardians of individuals less than 18 years of age. This research was also approved, as part of the EC NIDIAG project, by the London School of Hygiene and Tropical Medicine Ethics Committee as well as by the Ethics Committee of the Antwerp University Hospital.

### Sources of sera

A detailed description of all serum samples used in this work can be found below while a summary is shown in Table 1.

**Table 1:** Serum samples used in this study. Details of all serum samples used in the western blots, peptide arrays and RDT prototypes.

Sample type	Region	Definitions	n
Active VL	India	Samples were taken at or around onset of treatment (D0, D7 or D15)	25
Relapse		VL treated and subsequently relapsed to active disease. Sampled at the time of relapse diagnosis	26
		**Total India**	51
Active VL	Sudan	Samples were taken at or around onset of treatment (D0, D7 or D15)	19
NEHC	France	Samples from healthy donors living in region where no transmission of VL is documented	10

#### India

Indian sera were selected from archived samples, collected after 2007 from active VL, relapsed and endemic healthy controls (EHCs), all from the endemic region of Muzaffarpur, Bihar. Active VL cases had been diagnosed by positive rK39 and/or DAT serology and parasitologically by microscopy of splenic aspirates prior to the present study. Relapses were diagnosed clinically. All Indian samples were HIV negative.

#### Sudan

Sudanese serum samples were collected in 2011 and 2012 from the VL-endemic region of Gedaref in eastern Sudan. VL cases had been diagnosed by microscopy of bone marrow or lymph node aspirates in conjunction with serological assays (rK39 or rK28) and were all HIV negative. These diagnoses were carried out according to their respective national procedures, prior to the present study.

#### NEHC

Sera was obtained from whole blood collected from *Etablissement Français du Sang*, France. All donors were informed of the use of the blood for research purpose and gave their informed consent for the purpose of scientific research use and that all national laws and ethical principles were fulfilled. The samples were fully anonymised. All samples were certified to be negative for the following transmissible diseases: HIV-1, HIV-2, HCV, HBV, HTLV I, HTLV II and syphilis.

### Wet lab identification of *L. donovani* antigens detected by IgG1

*L. donovani* promastigote antigens recognised by human IgG1 were profiled using western blots as described in the repository protocols.io (dx.doi.org/10.17504/protocols.io.u8rezv6). Briefly, the cytosolic proteins from a *L. donovani* (strain MHOM/IN/80/DD8) whole cell lysate were separated by SDS-PAGE, blotted onto a nitrocellulose (NC) membrane and sliced into individual strips, to be immunoassayed with sera from individual patients from India. Upon visual identification of bands of interest on the individual strips using HRP-labelled anti-human IgG1 as secondary antibody, new gels were run and corresponding immunogenic bands were excised and analysed by mass spectrometry (MS) (syn. liquid chromatography tandem mass spectrometry (LC-MS/MS)), according to the methods described in S1 Text. Protein hits were identified by matching peptide fragments against the *L. donovani* reference genome-derived proteome only (ENA accession nos. FR799588-FR799623 [17]), henceforth referred to as LdBPK v1 genome. These proteins were submitted to the *in silico* filter detailed below.

### *In silico* refinement of wet lab output

**Fig 1:**
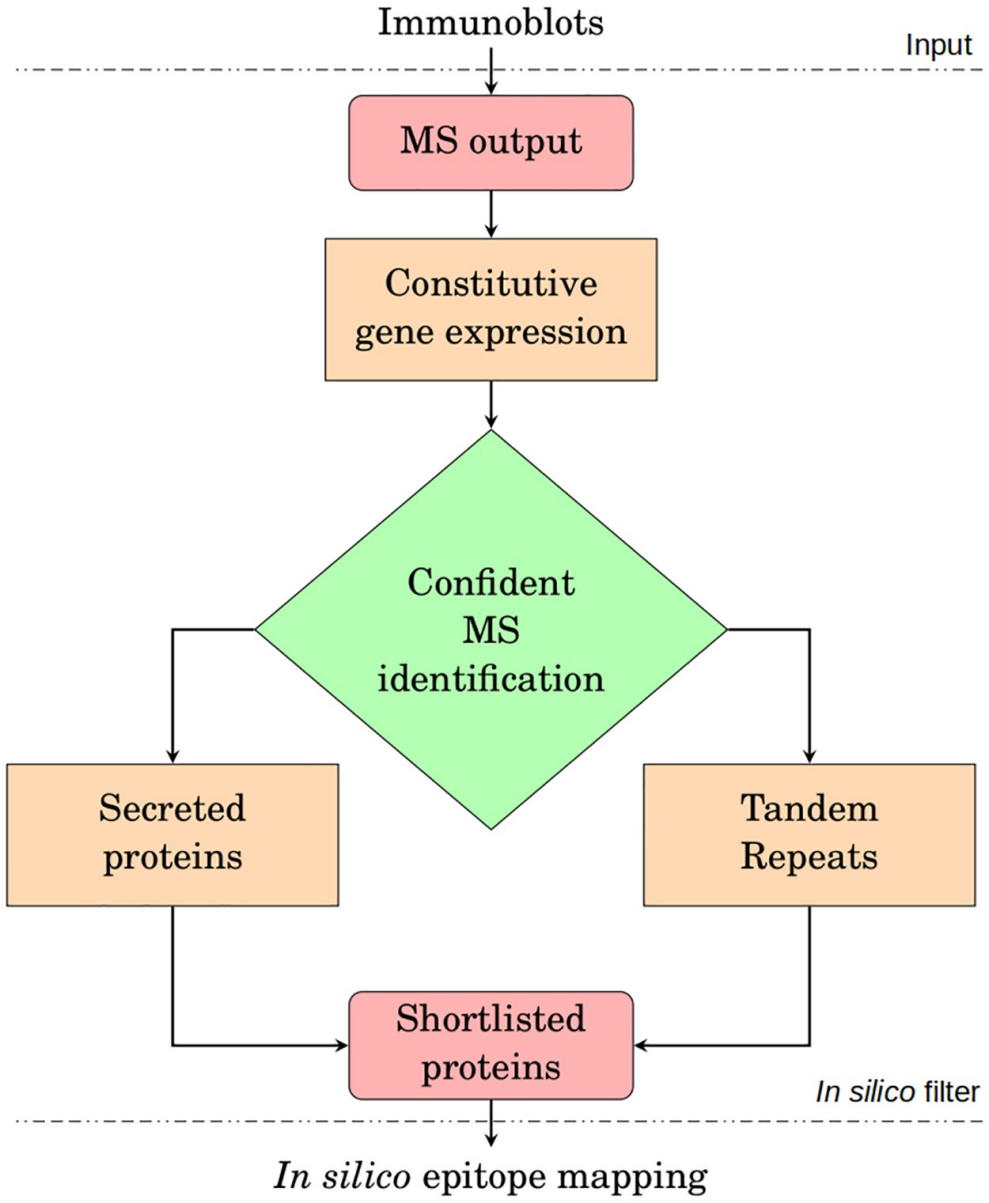
*In silico* filter applied to select desired protein features. Selected protein properties (yellow and green nodes) were searched *in silico* on all protein hits identified by MS from selected IgG1 sero-reactive western blot bands (*MS output*—top node), with the ultimate goal of shortlisting protein candidates most likely to be antigenic (*Shortlisted proteins*—bottom node).

Desired protein features were searched *in silico* in order to decrease the number of candidates from the MS output while shortlisting the proteins more likely to be antigenic, to have their B-cell epitopes mapped with *in silico* prediction algorithms. Some protein features were searched in series while others in parallel, meaning that all shortlisted proteins satisfy the criteria described in either branch as shown in Fig 1. These shortlisted v1 IDs were then merged with the IDs from an improved LdBPK282 reference genome (ENA accession number ERP022358, henceforth referred to as LdBPK v2) according to the correspondence in Data Set 2 from [30]. Hypothetical proteins were excluded, unless containing tandem repeats (TRs). Additional information on each step of this *in silico* filter are detailed in the following subsections.

#### Gene expression analysis—RNA-seq

Transcriptomic data on seven promastigotes and four amastigotes, representing different parasite life stages was obtained. In the present study the improved LdBPK282 reference genome was used [30]. *Leishmania* chromosome copy number is known to be variable, especially in cultivated promastigotes, which can potentially change within a few passages. To quantify the RNA-seq depth levels, which were not affected by somy variability, we used normalised haploid depth (HAP). The average normalised HAP of all genes were ranked for promastigotes and amastigotes separately and only those genes whose values were greater than the first quartile (HAP>Q1) in both parasite life stages were considered as constitutively expressed and and kept for downstream analysis.

#### Confident MS identification

We matched the peptides identified by MS against the LdBPK v1 genome only. Each protein hit identified by MS with the software MASCOT [31] has its own score defined by:

$$\begin{array}{c} \text{MASCOT}\;\text{score}=-10\times {\text{log}}_{10}\left(P\right) \end{array}$$

The score converted the probability that the observed match was not a random event (P) into an ascending scale where the lowest score is the most unreliable match, and higher scores indicate more reliability [32]. A score of 100 was chosen as the cut-off value to minimise the chances that any match could be found by chance.

#### Protein localisation

Exported *L. donovani* proteins, identified as part of exosomes by experimental approaches [33] and/or by the presence of a signal peptide were considered highly accessible to B-cells and thus selected for further analysis. The presence of a signal peptide sequence was searched with the stand-alone version of the software SignalP v4.0 [34] with the option ‘noTM’. All other optional parameters were default.

#### Antigenic domain selection

*L. donovani* proteins harbouring TRs include known B-cell antigens such as rK39 and rK28, therefore the presence of this domain was included as a criterion correlating with antigenicity. The protein coding DNA sequences were scanned for the presence of TRs using the stand-alone version of the program Tandem Repeats Finder [35] (v4.09). All program parameters were default.

### Epitope mapping from shortlisted proteins

Four *in silico* algorithms were used independently to define B-cell epitopes: ABCPred [23], Bepipred1.0 [22], EpiQuest-B [36] and lbtope [37]. For all epitopes predicted from each algorithm individually (generally a 15 or 16mer), a ‘core sequence’, stretching from the fifth to the eleventh AA residue was defined and scanned against the rest of the predicted antigenic peptides, in order to identify peptides of similar sequences. These peptides sharing core sequences with other predicted peptides at any position in the AA sequence are henceforth referred as partially overlapping peptides.

While ABCPred, lbtope and EpiQuest-B were run on default values, predicting antigenic sequences of 16, 15 and 16 AA residues, respectively, shorter sequences with high antigenic scores were also considered for EpiQuest-B. The highest scoring peptides predicted from Bepipred were obtained by calculating the area under the curve (AUC) from plots of AA residue position vs. individual residue score for all possible combinations of 16mers with one AA offset, considering all contiguous predicted antigenic sequences from all submitted proteins. Additional details about the prediction algorithms employed can be found in Table 2.

**Table 2:** Information on the B-cell epitope prediction algorithms employed. A total of 80 peptides with high antigenic scores, independently predicted from four different B-cell prediction algorithms, were selected for synthesis. AA: amino acid; AUC: area under the curve.

Algorithm	Details	n
ABCPred	Predicts antigenic regions of fixed even length and assigns a score, ranging from zero to one, for the whole predicted k-mer.	20
lbtope	Scans all possible 15mers with one AA offset on all the submitted sequences. A numeric score as well as the probability of correctnesses of each prediction are outputted.	20
EpiQuest-B	Calculates individual scores per AA residue (AGR) and the final peptides scores are ranked by the relative immunogenicity index, which corresponds to the curve (AUC) in a plot residue position vs. AGR.	20
Bepipred	Assigns a score for each AA residue on the submitted sequences. Predicted epitope regions can be of any length ≥ 1 AA with a score greater than the set cut-off (0.35 for default values). The antigenicity of the 16mers was calculated from the AUC in a plot residue position vs. score.	20
	**Total**	80

### Peptide synthesis

Desalted peptides i.e. of varying purity grades were synthesised with an N-terminal biotin molecule linked via AAG spacer so that they could bind to NC membranes (1620215, Bio-Rad, USA), which had been previously soaked with neutralite avidin (NLA) (NLA30, e-proteins, Belgium) and dried at 50°C. Lyophilised peptides were dissolved according to standard protocols [38]. Where the solvent mixture in which the peptides were dissolved exhibited stable background values, the peptide concentration was calculated based on their molar absorbances, measured either at 280 nm, for tyrosine (Y) and tryptophan (W) containing peptides [39], or at 205 nm [40], for those without W or Y. For the remaining peptides, the concentration was calculated by dividing the peptide amount reported by the manufacturer by the volume of the solvent mixture in which they were dissolved.

Any promising candidate identified from the pilot peptide screening was synthesised with higher purity grade (>90%) and with a N-terminal biotin—polyethylene glycol-glycine spacer in order to improve water solubility as well as to increase rotation and ensure that the full amino acid sequence could freely interact with Igs, as opposed to being adsorbed onto the solid support and therefore unavailable for recognition by Ig [41].

### Pilot screening of peptides using arrays

#### Array production

sciFLEXARRAYER (Scienion AG, Germany) with a PDC 70 piezo dispense capillary (type 3 coating, P-2030/ S-6051, Scienion AG, Germany) was used to spot 7 nl of the selected peptides at the required concentrations in multiple replicates onto NC membranes previously soaked with NLA and dried at 50°C. rK39 (RAG0061, Rekom Biotech, Spain) and a whole *L. donovani* lysate, obtained as described in [29], were both spotted on the diagonals as positive controls as well as for orientation purposes. As negative controls, we spotted a peptide specific for *T. cruzi* [42]. All controls were spotted at 0.1 mg/ml. Arrays were incubated overnight (ON) at 50°C upon completion of the spotting, to be hybridised with serum the next day.

#### Hybridisation with serum/ image acquisition

Peptide arrays were blocked with phosphate-buffered saline (PBS) + 3% bovine serum albumin (BSA) (PBSB) ON at 4°C or for 2h at room temperature (RT), followed by three 5 minute washes of PBS + 0.05% Tween 20 (PBST). In order to assess the peptide recognition by sera from VL patients and NEHCs, separate arrays were hybridised with pooled Sudanese serum samples positive for VL and with commercial pooled NEHC sera (S1-100ML, EMD Millipore Corporation, USA), respectively, diluted in PBST + 3% BSA (PBSTB), for 1h. After five 5 minute washes with PBST, they were incubated with fluorescent mouse anti-Human IgG1 Fc—Alexa Fluor 488 antibody (AB) (A-10631, ThermoFisher) diluted 1:1000 in PBSTB, for 1h at RT. Followed by five 5 minute washes with PBST, the arrays were incubated at 50°C until completely dry.

Images were acquired at 500ms and 20 dB gain with a digital CCD camera (ORCA-R^2^, Hamamatsu, Japan) coupled to a fluorescence microscope (model BX53, Olympus, Germany) equipped with filter cube U-FGFP (N271350, Olympus, Germany). The fluorescence of the spots was quantified using the software cellSens Dimensions v.1.7 (Olympus GmbH, Germany)

### Adaptation to an RDT

RDTs were composed of a NC strip sensitised with NLA at 3.5 mg/ml and a conjugate pad, impregnated with anti-human IgG1-specific antibody conjugated to 40nm gold beads (nanoQ, Belgium). The strip was either housed within a plastic cassette, with a buffer application well and a test/reading window (cassette), or not (dipstick). Prior to application on the RDT, equal volumes of serum and the biotinylated peptide at stock concentration were mixed and incubated at 37°C for 15 minutes. 3.5 μl of the mix was then pipetted onto the sample application zone, just above the top of the sample pad and at the bottom end of the NC strip. 150 μl of buffer solution was dispensed into the buffer application well (cassette) or dipsticks were dipped into a recipient vessel filled with same volume of running buffer. After 15 minute incubation, a test was deemed valid if a clear red control band was present in line with the ‘C’, and deemed positive if a second band was present in line with the ‘T’. If no band was visible at the ‘T’ and a clear control band at ‘C’ developed, then the test was deemed ‘negative’. Invalid tests were assessed by the absence of the red control band ‘C’ or by any other clear migration problem.

### Statistical analysis

All statistical analyses were performed using the computing environment R [43]. The final fluorescence of each spot on the peptide arrays (spot fluorescence) was expressed in arbitrary units and calculated by subtracting the mean background value of a given acquired image from the individual fluorescence values. Mean background value was calculated from the mean of six-eight fluorescence measures in random positions on the arrays where no fluorescence signal was detected. The peptide specificity was expressed as 95% Fiellers confidence intervals (CIs) of the ratio between the mean spot fluorescence of eight replicates from the arrays hybridised with VL serum and that of arrays hybridised with NEHC serum (ratio VL/NEHC). CIs were calculated with the mratios R package [44].

RDT results were compared with defined clinical status to establish sensitivity and 95% CIs with Clopper-Pearson exact method using the PropCIs R package [45].

## Results

### MS of selected antigenic bands identified over 1300 hits

**Fig 2:**
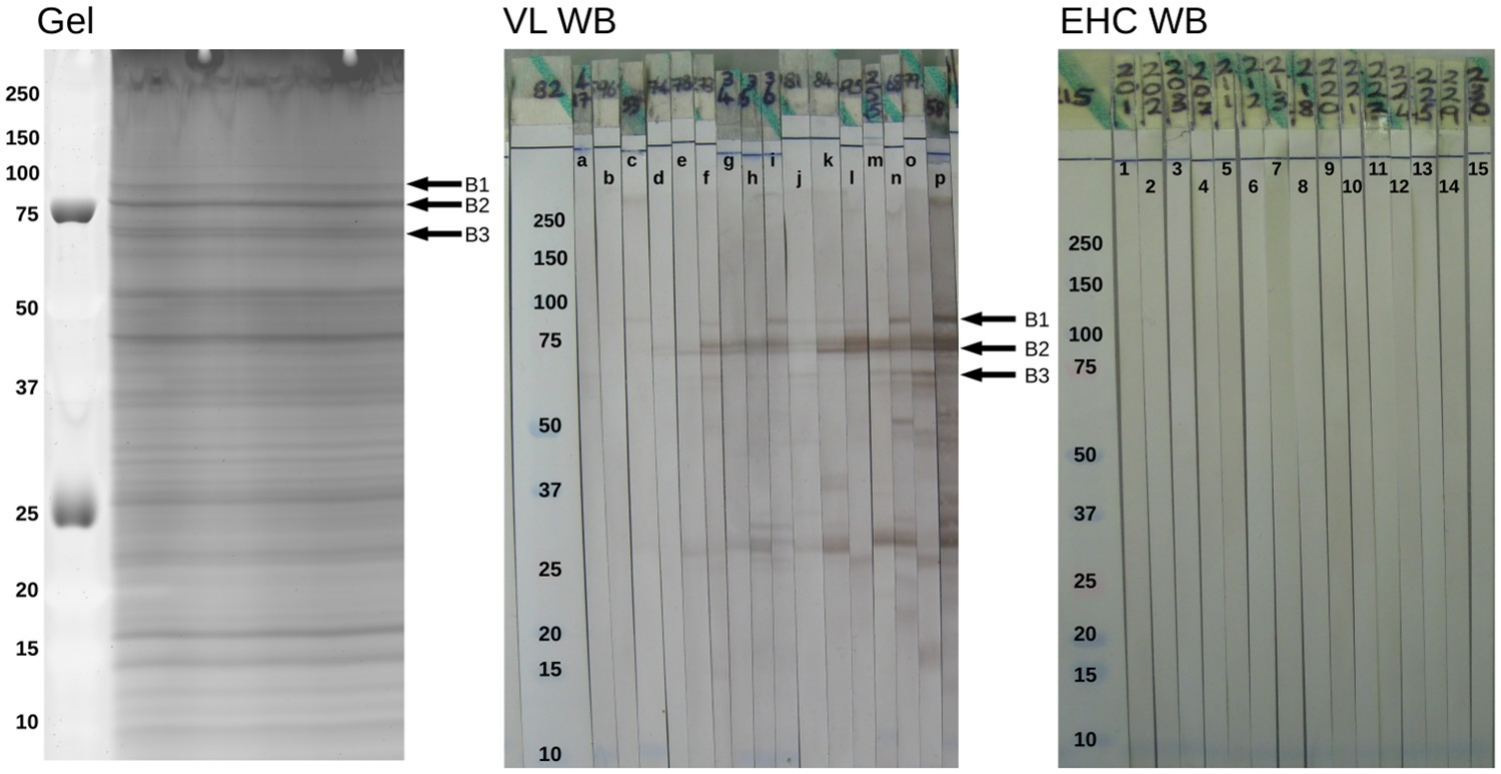
Gel and western blot strips immunoassayed with sera from Indian VL and EHC patients. The western blot strips incubated with individual active VL sera revealed the *L. donovani* protein bands reacting with IgG1 from Indian patients (VL WB). Three antigenic bands, B1, B2 and B3 were excised from gels to have their constituent proteins identified by MS (Gel). The strips incubated with EHC sera did not develop any band (EHC WB). Molecular mass markers are given in kDa.

The development of the western blot strips immunoassayed with active Indian VL serum samples and relapsed patients revealed the protein bands detected by human IgG1. New gels were run in order to get three selected antigenic bands (B1, B2 and B3) excised (Fig 2), to ultimately have their constituent proteins revealed by MS. Criteria to select the antigenic bands were (I) strong recognition, visually assessed by their intensity, (II) ubiquitous recognition i.e. recognised by the majority of the patients and (III) confident match between the bands from the western blots and the new gels.

A total of 1357 putative *L. donovani* proteins were identified from the three selected antigenic bands, with some identified in multiple bands (redundant matches). All these candidate antigen proteins were submitted to the *in silico* filter (Fig 1) to reduce the number of promising candidates while restricting to those possessing protein features that correlate with antigenicity.

### The *in silico* filter excluded over 90% of the uniquely identified proteins

**Fig 3:**
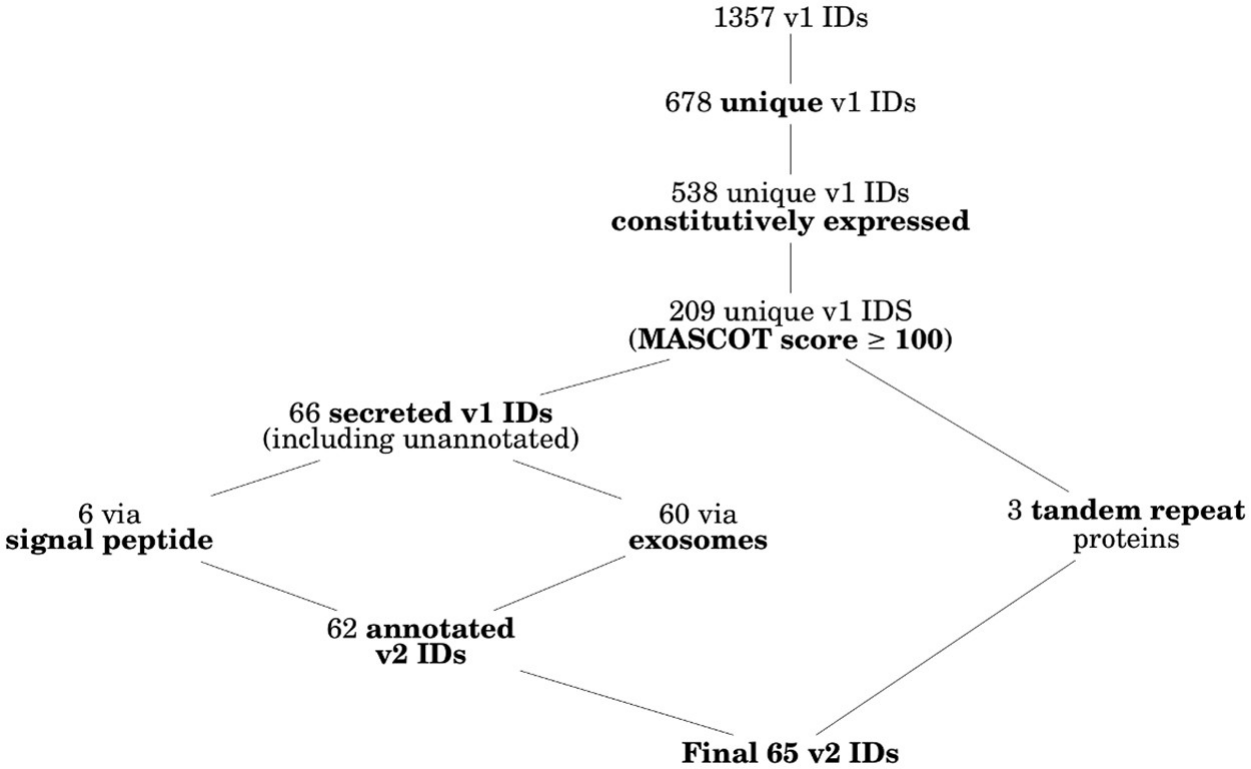
Selected proteins for *in silico* epitope mapping. 65 proteins (‘Final 65 v2 IDs’) satisfied the criteria in either branch shown in Fig 1 and were shortlisted to have their epitopes mapped with multiple *in silico* B-cell prediction algorithms. In **bold** are the features added after each step of the filter.

The initial 1357 IDs from the three selected antigenic bands (Fig 2) contained 678 unique IDs that matched the LdBPK v1 genome (we only matched the peptides identified by MS against the LdBPK v1 genome, such that the analysis returned *L. donovani* protein hits only). Out of these, 538 were considered to be constitutively expressed in both amastigote and promastigote life stages following gene expression analysis, of which 209 had a MASCOT score ≥ 100. Sixty six of these candidates were found to be secretory, 60 of which as part of exosomes while 6 where actively secreted to the outside of the cell via the presence of a signal peptide. These 66 unique v1 IDs were merged with the corresponding IDs from the improved LdBPK v2 reference genome, making 62 annotated extracellular and constitutively expressed proteins, identified with confidence by MS, as shown in Fig 3.

Further three unique candidates harbouring TR domains were found to be constitutively expressed and were identified with confidence by MS from the excised bands. Although none of the three was found to be extracellular, they were kept for downstream epitope mapping as such proteins containing TR domains are often B-cell antigens [46]. A single TR protein satisfying all the criteria described remained unannotated in the improved LdBPK v2 reference genome. Results including the number of hits satisfying all the criteria applied are schematically shown in Fig 3 while the detailed list with all the 65 shortlisted proteins can be found in S1 Table.

### *In silico* selection of B-cell epitopes

All 65 shortlisted proteins were profiled with the B-cell prediction algorithms as detailed in the methods section. Considering partially overlapping peptides, only that of highest score was shortlisted. The top 20 scoring peptides, with no shared core sequences within a given algorithm, were selected for synthesis, making a total of 80 high scoring peptides from four different prediction algorithms, as shown in S2 Table.

### Peptide antigenicity screening

#### Initial hybridisation revealed the most antigenic peptide

**Fig 4:**
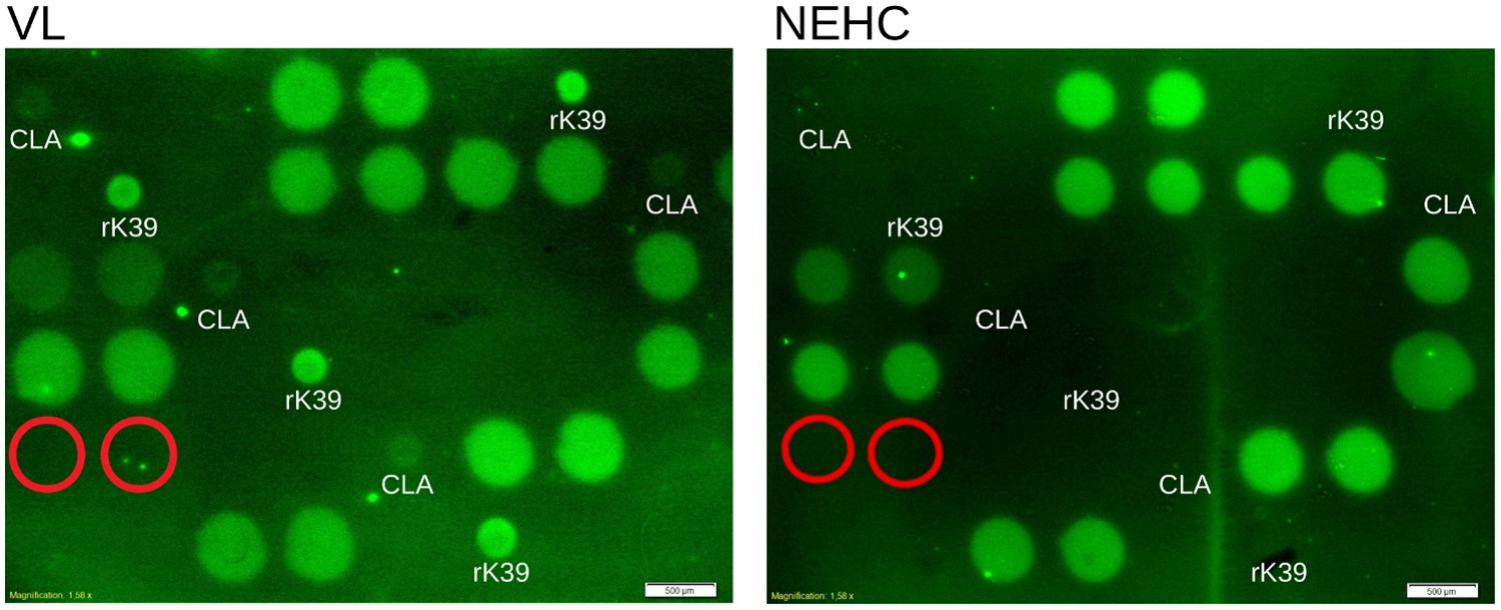
Peptides reactivity with a pool of Sudanese VL and NEHC sera diluted at 1:100 showed to be unspecific. [VL]: Section of an array hybridised with pooled Sudanese VL serum at 1:100 dilution. rk39 and a whole *L.donovani* lysate (CLA) were spotted on the diagonal as positive controls as well as for orientation purposes. [NEHC]: the same array section hybridised with a pool of NEHC sera at same dilution. The red circles indicate the spotting position of a peptide specific for *T. cruzi* [42], employed here as negative control. Both images indicate the array section where the peptide epitopes predicted from the lbtope algorithm were spotted.

A first batch of peptide arrays was produced in order to obtain insights about the reactivity of the peptides with human IgG1. The visual inspection of the imaged arrays hybridised with both pooled serum groups (Sudanese VL and NEHC) diluted 1:100 revealed that a high number of peptides spotted at their stock concentrations reacted with both serum pools (Fig 4).

**Fig 5:**
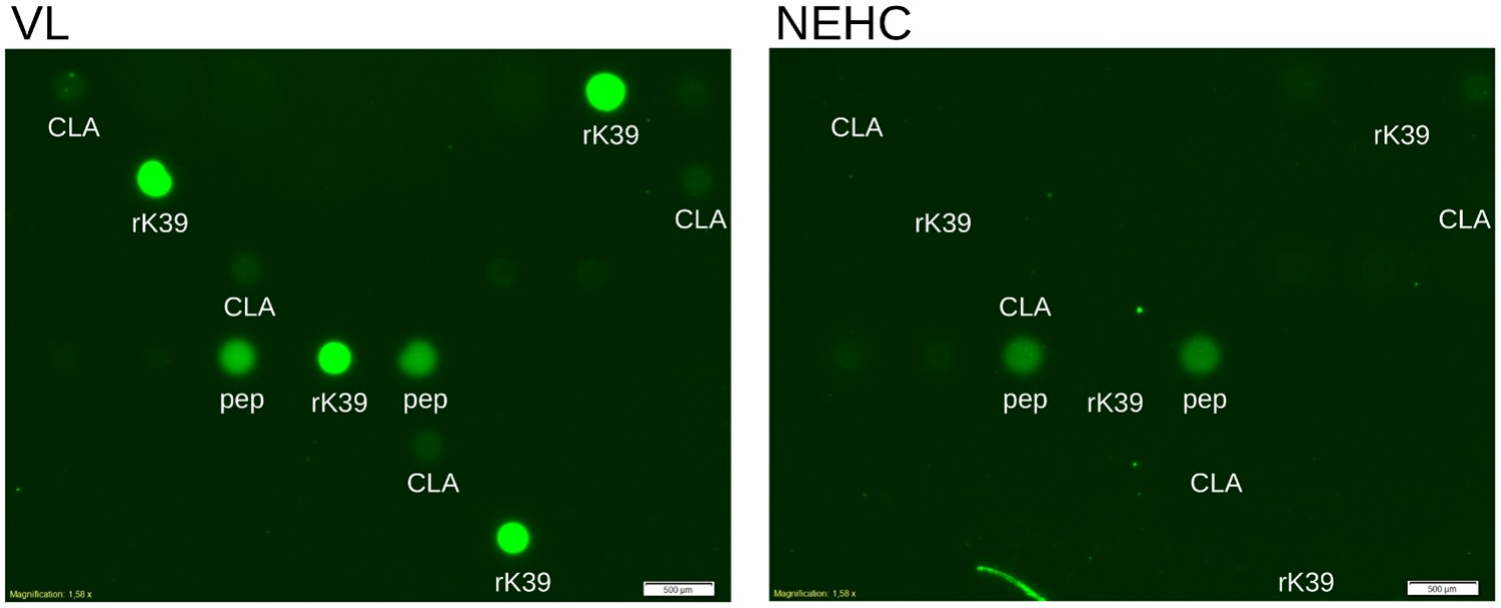
Hybridisation of the peptide arrays with a pool of serum samples diluted 1:200 revealed the most sensitive peptide. [VL]: Section of a peptide array hybridised with pooled Sudanese VL serum at 1:200 dilution. The most reactive peptide (pep) was spotted in duplicates. rk39 and CLA spotting positions are shown. [NEHC]: the same array hybridised with a pool of NEHC sera at same dilution. Both images are from the array section where the peptide epitopes predicted from the EpiQuest algorithm were spotted.

Hybridisation of the same batch of arrays with pooled serum at a higher dilution (1:200) showed that a reduced number of peptides reacted with both serum groups, visually revealing the most reactive peptide, as shown in Fig 5. Details of the most reactive peptide found, henceforth referred as EpQ11, are shown in Table 3.

**Table 3:** Information on the most antigenic peptide identified visually from the pilot screening with desalted peptides. Column ‘v2 ID’ and ‘v2 gene product’ refer to the improved LdBPK reference genome.

Peptide ID	Short ID	Sequence	AA residues	v2 ID	v2 gene product
EpQ_11_NIRI	EpQ11	NIRIHLGDTIRIAPCK	82—97	LdBPK_360019900.1	Transitional endoplasmic reticulum ATPase, putative

#### Specificity of peptide EpQ11 was highly concentration dependent

**Fig 6:**
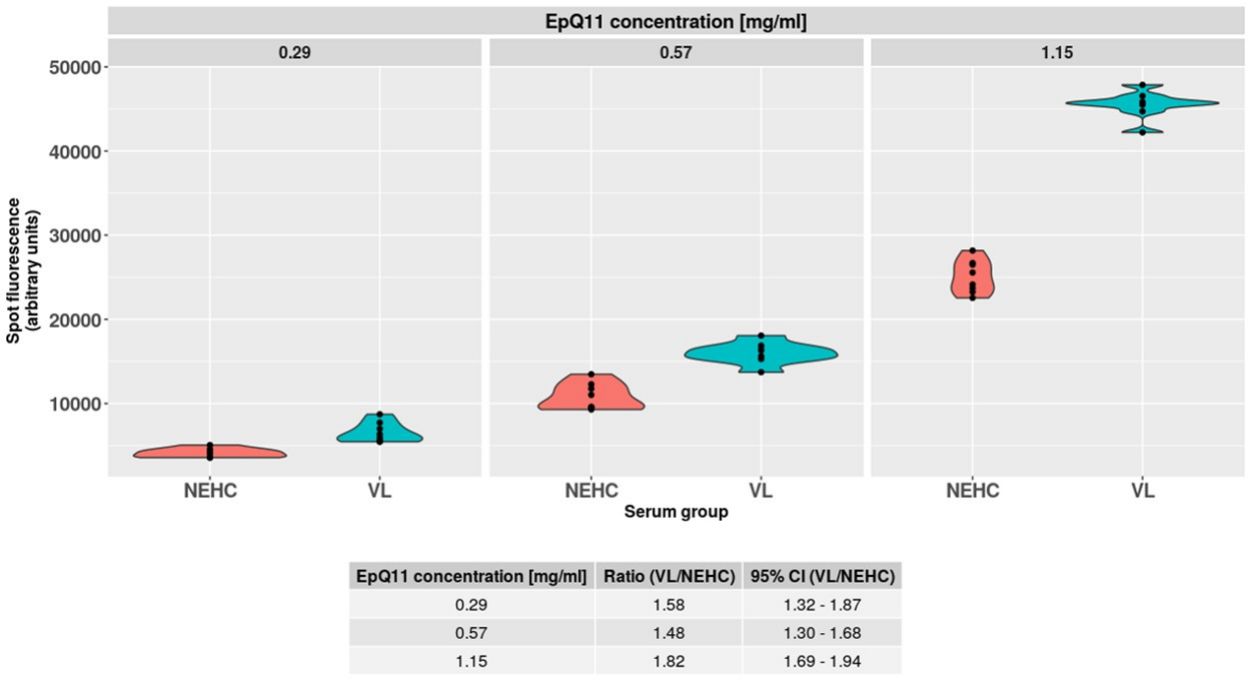
Peptide specificity for VL IgG1 was highly concentration dependent. Peptide EpQ11 was spotted at three different concentrations in eight replicates each onto dry NC membranes, previously soaked with NLA. The highest discrimination between VL and NEHC IgG1 (Ratio VL/NEHC) was obtained when the peptide was spotted at 1.15 mg/ml.

The most reactive peptide from the initial pilot screening, EpQ11, was spotted at multiple concentrations in eight replicates each onto a NC membrane pre-soaked and dried with NLA at 1 mg/ml and dried. The hybridisation of two separate arrays with pooled serum samples diluted 1:200 showed that the discrimination between the VL and the NEHC pooled sera was highly dependent on peptide concentration, with the highest discrimination displayed when the peptide was spotted at 1.15 mg/ml, as shown in Fig 6.

#### Peptide EpQ11 bound specifically to IgG1 from VL patients in RDT prototypes

**Fig 7:**
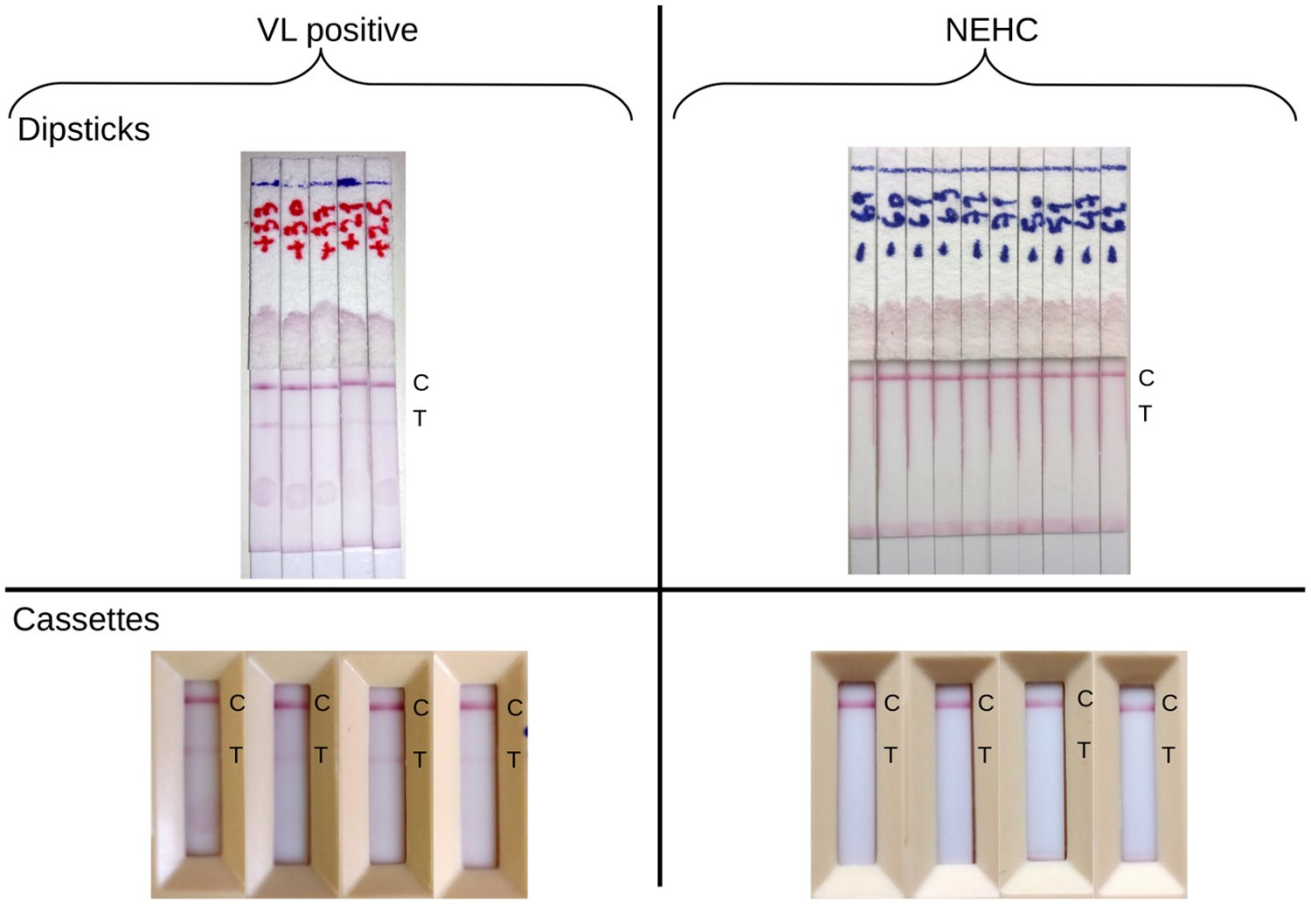
Peptide EpQ11 is specifically detected by Sudanese VL IgG1 in cassette as well as in dipstick format. ‘T’ indicates the location of the NLA at which, in a positive test, there is a red coloured line due to the presence of peptide-IgG1 complex. Successful migration was ensured by the development of the control line at ‘C’.

RDT prototypes in two formats, cassettes and dipsticks, were tested with individual Sudanese VL serum samples as well as NEHC from Europe, in order to confirm the specificity of the EpQ11 peptide in a point-of-care format. All of the NEHC sera (n = 10) were negative by both RDT formats. Sensitivity values, assessed with VL serum samples (n = 19) varied between 79% (54—94%) and 84% (60—97%) depending on the format tested (Table 4). Representative results for these RDTs are shown in Fig 7.

**Table 4:** EpQ11 performance in RDTs with Sudanese VL and NEHC patient sera. Summary of the RDT prototypes tested with individual VL sera from Sudan or NEHC serum samples from Europe.

Serum Group	n	Format	RDT results	
			Positive	Negative
VL positive	19	Cassette	84,2% (16/19)	15.8% (3/19)
		Dipstick	78.9% (15/19)	21.1% (4/19)
NEHC	10	Cassette^a^	0% (0/8)	100% (8/8)
		Dipstick	0% (0/10)	100% (10/10)

^a^: 2/10 tests were deemed invalid (control line did not develop due to leakage of running buffer from the buffer application well) and were therefore excluded from the sensitivity calculation.

## Discussion

Identifying a biomarker of post-chemotherapeutic relapse remains a key element for VL control worldwide. The majority of relapses occur between 6 and 12 months after therapy completion [47], while rates are drug dependant and can reach levels up to 20% at 12 months after completion of therapy in immunocompetent patients [48]. The situation is even more dramatic in HIV-coinfected patients, where relapse rates as high as 70% have been reported [49]. Considering that most of the patients affected by VL live in poor and remote villages in developing countries [50], the development of easy-to-use diagnostic tools is of utmost importance in order to follow up patients up to 12 months upon treatment completion in a primary care setting. Currently there is no point-of-care serodiagnostic tool for detection of relapses in VL. Despite unquestionable value of the rK39-based RDTs for active cases identification, they cannot be used as a tool to detect relapses due to antibody persistence even after complete disease clearance [9–13].

We have identified a peptide detected by human IgG1 from VL patients by mapping epitopes from multiple proteins, which were selected from immunoblots by searching antigenic features using comparative genomics and *in silico* algorithms. The peptide was adapted to RDT prototypes and showed promising results for VL serology in terms of both sensitivity and specificity with limited sample size. Given the free online availability and growing number of various *in silico* algorithms to predict or scan multiple protein features from DNA or AA sequences and the growing number of high quality sequenced genomes, the adopted strategy can also be employed for the search of diagnostic biomarkers or vaccine candidates for other infectious diseases [51, 52].

The decision to make use of comparative genomics and *in silico* algorithms to refine the output from immunoblots came from the excessive number of putative hits identified by MS, and we believe that the strength of our approach lies in this combination. Nonetheless, we consider that technical issues might have been related to the excessive number of hits initially identified by MS from the western blots. The immunogenic bands excised from the acrylamide gels that were analysed by MS were excised from gels with wide lanes (59 mm). We believe that excising such wide lanes from acrylamide gels contributed to the excessive number of hits: by mechanically cutting along very wide lanes, we have probably included proteins that would not have been present had we excised narrower (e.g. 4 mm wide) lanes, although multiple hits would have been probably identified anyway.

We have identified antigens from *L. donovani* promastigotes due to ease of culturing. In the host, promastigotes are phagocytosed by macrophages or other types of mononuclear phagocytic cells, where they differentiate into amastigotes, remaining in this form until transmission to a new vector [53]. Therefore we believe that amastigote proteins are more likely to be targeted by host Igs. We have chosen a non-stringent HAP cut-off for both promastigotes and amastigotes (HAP>Q1) due to the generally nonstrong correlation between mRNA and protein expression levels observed in eukaryotes [54, 55]. By doing so, we avoided missing antigens that might be constitutively expressed, despite low mRNA levels. Given the excessive number of protein candidates identified by MS we could set a stricter MASCOT score cut-off, ruling out proteins that could have been identified by chance in each antigenic band. Extracellular proteins are more accessible for binding with host Igs, thus we have included this criterion in our filter. Finally, because intracellular proteins harbouring TRs are amongst the most widely used antigens for VL diagnosis [56], we decided to select such candidates in parallel to extracellular proteins. Although the vast majority of epitopes founds in proteins are discontinuous [57], the use of such prediction algorithms is only meaningful in case the native 3D structure of the proteins is retained, which is not the case upon completion of an SDS-PAGE gel. In addition, the prediction of discontinuous epitopes relies on the scarce availability of experimentally validated epitopes.

Even though the *in silico* filter that we applied ruled out over 90% of the proteins initially identified by MS from the immunoblots, it remained impracticable to test the antigenicity of all 65 selected proteins. By screening synthetic peptides instead of recombinant proteins we circumvented the lengthy and costly protein expression and purification steps and could therefore test a large number of top-scoring candidates predicted from multiple algorithms with various prediction methods. Moreover, the production of peptide arrays allowed the screening of multiple peptides at various concentrations using low volumes of reagents of limited availability to us (e.g. VL serum).

The pilot screening with peptide arrays revealed that the EpQ11 was the most sensitive peptide, despite cross-reacting with NEHC serum (Fig 5). By showing the clear effect of the hybridisation conditions on the specificity of the EpQ11 in arrays, especially that of the spotting concentration (Fig 6), we sought to test the EpQ11 in RDTs, taking into account that the format of the test (i.e. peptide array vs. RDTs) could have an even larger impact on the specificity/ sensitivity of the peptide. We believe that the long hybridisation periods employed in our peptide arrays played a crucial role on the higher sensitivities observed when compared to the RDT prototypes. On the other hand, by using a format of lower sensitivity, we managed to avoid the false positive signals generated by the NEHC samples in peptide arrays. The intensity of the test line of our RDT prototypes might be improved by using a larger peptide composed of multiple copies in series (‘peptide trains’) or in parallel (multiple antigen peptides), a strategy already used in vaccine development [58, 59], to boost the recognition of synthetic peptides by Igs, although we are aware that this improvement in sensitivity might have a negative effect on the specificity of the test.

In spite of these advantages of screening peptide epitopes in a high-throughput manner, the most important antigens for VL diagnosis (e.g. rK39, rK28) are still recombinant proteins. It would have been an interesting alternative to apply the *in silico* filter described here to proteins of lower molecular weight (e.g. those between 25 and 37 kDa—Fig 2), to test the antigenicity of a few of them. Low molecular weight proteins are generally easier to express and purify when compared to large antigens. We decided, however, to focus our search in predicted epitopes from larger proteins due to (I) the large number of possible epitopes that proteins of high molecular weight have and (II) we were not entirely convinced about the matching between the antigenic bands in the western blots and those in the gels, mainly due to the high density of proteins in that region of the gel (Fig 2).

**Fig 8:**
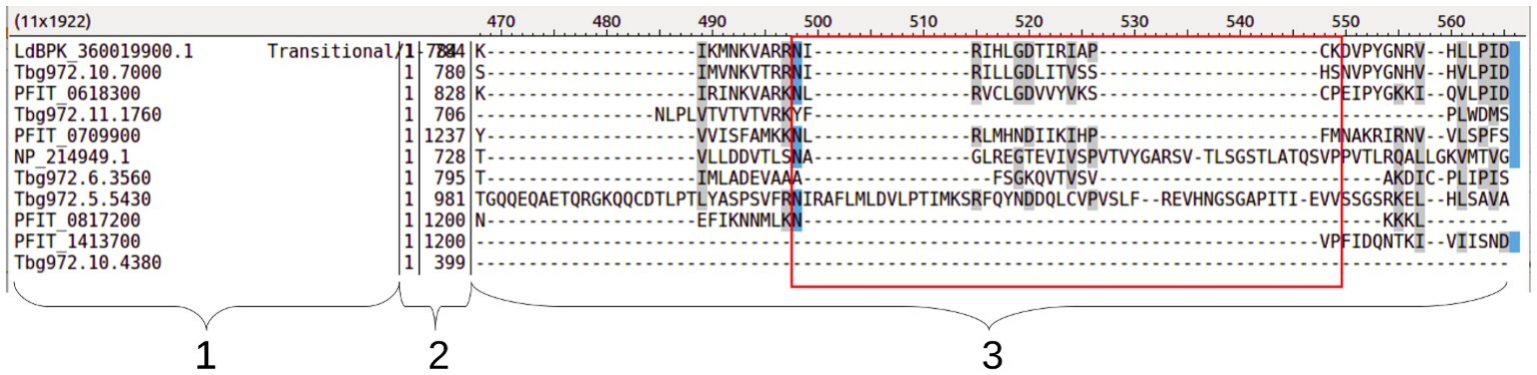
The *L. donovani* protein containing the EpQ11 sequence shows low similarity to proteins from organisms causing potentially cross-reacting diseases. The *L. donovani* protein harbouring the EpQ11 peptide (LdBPK_360019900.1—v2 ID) was aligned against the proteome of organisms causing potentially cross-reacting diseases with VL using MAFFT v7.222 [61]. Column 1: GenBank accession number; column 2: protein length (position of first amino acid | position of last amino acid, 784 for LdBPK_360019900.1), column 3: alignment around the region where the EpQ11 peptide is located. Numbers on top (470-560) indicate the alignment position. Screenshot from belvu, SeqTools—4.44.1 [62] showing the most similar proteins to LdBPK_360019900.1.

Another limitation of our study lies in the restricted range of serum samples available to us. Our ultimate goal was to use the EpQ11 peptide to detect IgG1 from pre-treatment VL patients paired with post-treatment (deemed cured), such that we could assess its use as a replacement for the *Leishmania* CLA in IgG1 RDTs, as described in [29]. Replacing the CLA by synthetic peptides would allow the manufacturing of more robust RDTs as a non-invasive tool to help in supporting the confirmation of cure after successful chemotherapy. Advantages of employing synthetic peptides (vs. whole-cell lysates) include unproblematic and low-cost synthesis and the production of more standardised (and potentially more sensitive and specific) diagnostic tests. Due to local regulations, however, it is not possible to export human sera from India, where the cured paired serum samples from our partner institutions are available (western blots were carried out on site in 2015), such that we could only carry out the peptide pilot screening as well as the sensitivity tests in RDTs with Sudanese active VL sera. As, however, Sudanese patients show generally a weaker immune response when compared to Indian VL patients [60], the serum samples that we tested can be considered ‘worst-case scenario’ samples such that we would expect the sensitivity in India to be at least as high as the values reported in this study (Table 4). The EpQ11 was also not tested with EHCs. However, EHCs used in both western blots (Fig 2) and ELISAs were negative with *L. donovani* CLA [29]. Finally, even though it is important to test the EpQ11 with serum samples from patients presenting with potentially cross-reacting diseases (e.g. malaria, human African trypanosomiasis), we would not expect a high rate of false positives due to low protein sequence conservation between the LdBPK_360019900.1. and proteins from organisms causing potentially cross-reacting diseases, especially in the region harbouring the EpQ11 sequence (Fig 8).

Our work was conducted as a proof of concept that comparative genomics and *in silico* algorithms can be employed downstream of wet lab experiments for a rational search for diagnostic peptides. Further investigations, using a larger sample size of various clinical status, including cured paired samples, EHCs as well as sera from patients presenting with potentially cross-reacting diseases, are still required to confirm the potential of the EpQ11 (with possible structural optimisations described above) as a stand-alone antigen for VL serodiagnosis. Meanwhile, given the evidence from the present work that the described peptide specifically binds to IgG1 from Sudanese VL patient sera, the sequence of the EpQ11 could be synthetically coupled to that of the rK39, potentially improving the sensitivity of the latter antigen, including in IgG1 RDTs, whose potential in monitoring clinical status in VL has recently been shown [63].

### Conclusion

Based on the interpretation of the results of our experiments we conclude that:

1. Comparative genomics as well as *in silico* algorithms are useful tools for refining large output from wet lab experiments for a rational search for diagnostic peptides.
2. B-cell epitopes prediction algorithms represent an interesting option for epitope mapping, enabling the screening of peptides in a high-throughput manner with minimal reagent consumption.
3. We have identified a peptide that specifically binds to human IgG1 from Sudanese VL patients by refining wet lab experiments with *in silico* searches. Replacing the CLA by specific antigens would enable the manufacturing of more robust IgG1 RDTs for monitoring clinical status in VL.
4. While further investigations are still required to confirm the potential of the EpQ11 peptide as a stand-alone antigen for VL serodiagnosis, incorporation of its sequence into that of rK39-based assays (i.e. rK39-EpQ11) might boost the sensitivity of this antigen in eastern African patients as well as in IgG1 RDTs.

## Supporting information

S1 Text LC-MS/MS method employed for protein identification. (PDF)

S1 Table Shortlisted proteins for *in silico* epitope mapping. List of 65 proteins identified by immunoblots and mass spectrometry (MS), then shortlisted by *in silico* algorithms. NCBI GenBank accession numbers are given for v1 IDs. Mass is given in kDa.
(PDF)

S2 Table Synthesised peptides for antigenicity screening in arrays. The top 20 scoring peptides from each of the four prediction algorithms were shortlisted and synthesised for antigenicity screening with Sudanese VL serum samples. ‘C.reported’ was calculated by dividing the reported amount by the volume of the solvent mixture in which the peptides were dissolved, while ‘C.280’ and ‘C.205’ were calculated based on the molar absorbances at 280 and 205 nm, respectively. All concentrations are given in mg/ml. MW is given in g/mol. Peptides that could not be dissolved and were therefore not included in the pilot peptide screening are marked with an asterisk.
(PDF)
