# Supplementary material for: Refining wet lab experiments with in silico searches: A rational quest for diagnostic peptides in visceral leishmaniasis
Source: PLoS Negl Trop Dis. 2019 May 6;13(5):e0007353. doi: 10.1371/journal.pntd.0007353 (PMC6522066; doi:10.1371/journal.pntd.0007353)
Supplement: S1 Text — (PDF) [file pntd.0007353.s001.pdf]

Liquid Chromatography Tandem Mass Spectrometry*Method

April 17, 2019

1 Goal

This document details the employed liquid chromatography tandem mass spectrometry (LC-MS/MS) method used to identify *L. donovani* proteins from antigenic bands excised from gels, as described in `dx.doi.org/10.17504/protocols.io.u8rezv6`.

2 Method

Peptides were resuspended in 5% formic acid and 5% DMSO and were separated on an EASY-nLC 1000 UHPLC system (Proxeon) and electrosprayed directly into an Orbitrap Elite mass spectrometer (Thermo Fischer Scientific) through an EASY-Spray nano-electrospray ion source (Thermo Fischer Scientific). The peptides were trapped on an in-house packed guard column (75 µm i.d. x 500 mm, RSLC C18, 2 µm, 100 Å) using a linear gradient (length: 45 minutes, 8% to 30% solvent B (0.1% formic acid in acetonitrile), flow rate: 200 nL/min). The raw data was acquired on the mass spectrometer in a data-dependent mode using a CID based method. Full scan MS spectra (scan range 350-1500 m/z, resolution 30000, AGC target 1e6, maximum injection time 250 ms) and of 10 most intense peaks were fragmented and analysed in the ion trap. CID MS/MS spectra were acquired using an AGC target 5e3, maximum injection time 100 ms, normalized collision energy 35%.

The raw data files generated were processed using MaxQuant (Version 1.5.0.35), integrated with the Andromeda search engine as previously described (Cox & Mann, 2008; Cox et al., 2011). For protein groups identification, peak lists were searched agaist the reference genome-derived proteome (ENA accession nos. FR799588-FR799623 (Downing et al., 2011) database as well as list of common contaminants by Andromeda. Trypsin with a maximum number of missed cleavages of 2 was chosen. Acetylation (Protein N--term), Oxidation (M) and Phosphorylation (S,T and Y) were used as variablemodifications while Carbamidomethylation (C) was set as a fixed modification. Protein and PSM false discovery rate (FDR) were set at 0.01 and a minimum score of 40 and

*Throughout the manuscript, the acronyms MS and LC-MS/MS can be used interchangeably

localisation probability of >0.7. for phosphopeptides. Match between runs was applied.

References

Cox, J., & Mann, M. (2008). Maxquant enables high peptide identification rates, individualized ppb-range mass accuracies and proteome-wide protein quantification. *Nature biotechnology*, *26*(12), 1367.

Cox, J., Neuhauser, N., Michalski, A., Scheltema, R. A., Olsen, J. V., & Mann, M. (2011). Andromeda: a peptide search engine integrated into the maxquant environment. *Journal of proteome research*, *10*(4), 1794–1805.

Downing, T., Imamura, H., Decuypere, S., Clark, T. G., Coombs, G. H., Cotton, J. A., ... Berriman, M. (2011, December). Whole genome sequencing of multiple leishmania donovani clinical isolates provides insights into population structure and mechanisms of drug resistance. *Genome research*, *21*, 2143–2156. doi: 10.1101/gr.123430.111
