# Supplementary material for: Refining wet lab experiments with in silico searches: A rational quest for diagnostic peptides in visceral leishmaniasis
Source: PLoS Negl Trop Dis. 2019 May 6;13(5):e0007353. doi: 10.1371/journal.pntd.0007353 (PMC6522066; doi:10.1371/journal.pntd.0007353)
Supplement: S1 Table — List of 65 proteins identified by immunoblots and mass spectrometry (MS), then shortlisted by in silico algorithms. NCBI GenBank accession numbers are given for v1 IDs. Mass is given in kDa. (PDF) [file pntd.0007353.s002.pdf]

S1 Table. List of 65 proteins identified by immunoblots and mass spectrometry (MS), then shortlisted by *in silico* algorithms. NCBI GenBank accession numbers are given for v1 IDs. Mass is given in kDa.

v1 ID	Score	Mass	v1 gene product	SignalP	Exosomes	v2 ID	v2 gene product
LdBPK_283000.1	5140	71509	heat-shock protein hsp70, putative	no	yes	LdBPK_280035200	heat-shock protein hsp70, putative
LdBPK_360210.1	3987	94942	elongation factor 2	no	yes	LdBPK_360006900	elongation factor 2
LdBPK_261550.1	2463	77764	thimet oligopeptidase, putative	no	yes	LdBPK_260020900	thimet oligopeptidase, putative
LdBPK_361420.1	2039	87562	Transitional endoplasmic reticulum ATPase, putative	no	yes	LdBPK_360019900	Transitional endoplasmic reticulum ATPase, putative
LdBPK_141240.1	1917	46634	enolase	no	yes	LdBPK_140018000	enolase
LdBPK_171390.1	1388	81273	translation initiation factor, putative	no	yes	LdBPK_170021100	translation initiation factor, putative
LdBPK_050960.1	1360	76269	dipeptidyl-peptidase III, putative	no	yes	LdBPK_050014700	metallo-peptidase, Clan M-, Family M49
LdBPK_271230.1	1326	78777	arginyl-tRNA synthetase, putative	no	yes	LdBPK_270018800	arginyl-tRNA synthetase, putative
LdBPK_354780.1	1306	79464	hypothetical protein, conserved	yes	no	LdBPK_350053100	Hsp70 protein, putative
LdBPK_120255.1	898	88757	unspecified product	no	yes	LdBPK_120007600	cysteinyl-tRNA synthetase, putative
LdBPK_161510.1	895	69373	paraflagellar rod protein 2C	no	yes	LdBPK_160020100	paraflagellar rod protein 1D, putative
LdBPK_210890.1	794	84741	methionyl-tRNA synthetase, putative	no	yes	LdBPK_210013900	methionyl-tRNA synthetase
LdBPK_090820.1	763	83734	oligopeptidase b	no	yes	LdBPK_090013400	oligopeptidase b
LdBPK_181350.1	715	92595	heat shock protein, putative	no	yes	LdBPK_180018900	heat shock protein 110, putative
LdBPK_365870.1	712	126694	isoleucyl-tRNA synthetase, putative	no	yes	LdBPK_360066500	isoleucyl-tRNA synthetase, putative

S1 Table continued from previous page

v1 ID	Score	Mass	v1 gene product	SignalP	Exosomes	v2 ID	v2 gene product
LdBPK_180670.1	644	77642	hypothetical protein, conserved	no	yes	LdBPK_180011800	HEAT repeats, putative
LdBPK_362140.1	592	59647	chaperonin HSP60, mitochondrial precursor	no	yes	LdBPK_360027400	chaperonin HSP60, mitochondrial precursor
LdBPK_261220.1	585	70850	heat shock protein 70-related protein	no	yes	LdBPK_260017500	heat-shock protein hsp70, putative
LdBPK_281850.1	578	108378	proteasome regulatory non-ATP-ase subunit 2, putative	no	yes	LdBPK_280023700	proteasome regulatory non-ATP-ase subunit 2, putative
LdBPK_290790.1	537	86902	lipophosphoglycan biosynthetic protein, putative	yes	no	LdBPK_290012400	heat shock protein 90, putative
LdBPK_281310.1	498	71957	glucose-regulated protein 78, putative	yes	no	LdBPK_280017700	heat-shock protein hsp70, putative
LdBPK_321000.1	475	102138	hypothetical protein, conserved	no	yes	LdBPK_320015100	Staphylococcal nuclease homologue/Tudor domain containing protein, putative
LdBPK_291330.1	458	80059	hypothetical protein, unknown function	no	yes	LdBPK_290018200	Hsp70 protein/TPR repeat/Tetratricopeptide repeat, putative
LdBPK_366960.1	439	60979	2,3-bisphosphoglycerate-independent phosphoglycerate mutase	no	yes	LdBPK_360079000	2,3-bisphosphoglycerate-independent phosphoglycerate mutase
LdBPK_091020.1	420	51622	elongation factor-1 gamma	no	yes	LdBPK_090015600	elongation factor-1 gamma

S1 Table continued from previous page

v1 ID	Score	Mass	v1 gene product	SignalP	Exosomes	v2 ID	v2 gene product
LdBPK_210880.1	403	251023	hypothetical protein, conserved	no	yes	LdBPK_210013800	CCR4-NOT transcription complex subunit 1 TTP binding domain/CCR4-NOT transcription complex subunit 1 CAF1-binding domain/Domain of unknown function (DUF3819)/CCR4-Not complex component, Not1, putative
LdBPK_010790.1	398	45356	eukaryotic initiation factor 4a, putative	no	yes	LdBPK_010012800	eukaryotic initiation factor 4a, putative
LdBPK_362130.1	396	60859	chaperonin HSP60, mitochondrial precursor	no	yes	LdBPK_360027300	chaperonin HSP60, mitochondrial precursor
LdBPK_081020.1	386	62770	stress-induced protein sti1	no	yes	LdBPK_080016400	stress-induced protein sti1
LdBPK_353150.1	375	100128	ATP-dependent RNA helicase, putative	no	yes	LdBPK_350036600	ATP-dependent RNA helicase, putative
LdBPK_211330.1	373	60225	T-complex protein 1, delta subunit, putative	no	yes	LdBPK_210018500	T-complex protein 1, delta subunit, putative
LdBPK_353900.1	342	62296	T-complex protein 1, eta subunit, putative	no	yes	LdBPK_350044300	T-complex protein 1, eta subunit, putative
LdBPK_130090.1	332	57615	carboxypeptidase, putative	no	yes	LdBPK_130005800	carboxypeptidase, putative
LdBPK_242150.1	306	72575	transketolase	no	yes	LdBPK_240027000	transketolase
LdBPK_110640.1	300	36477	aminopeptidase, putative	no	yes	LdBPK_110011900	aminopeptidase, putative
LdBPK_353280.1	287	45068	cystathione gamma lyase, putative	no	yes	LdBPK_350038000	cystathione gamma lyase, putative
LdBPK_332670.1	252	57440	carboxypeptidase, putative	no	yes	LdBPK_330034900	carboxypeptidase, putative
LdBPK_364030.1	252	70980	glycyl tRNA synthetase, putative	no	yes	LdBPK_360047600	glycyl tRNA synthetase, putative

S1 Table continued from previous page

v1 ID	Score	Mass	v1 gene product	SignalP	Exosomes	v2 ID	v2 gene product
LdBPK_367240.1	251	58671	chaperonin, putative	no	yes	LdBPK_360081800	T-complex protein 1, theta subunit, putative
LdBPK_071020.1	223	42353	splicing factor ptsr1-like protein	no	yes	LdBPK_070014900	splicing factor ptsr1-like protein
LdBPK_231460.1	223	60848	T-complex protein 1, gamma subunit, putative	no	yes	LdBPK_230020700	T-complex protein 1, gamma subunit, putative
LdBPK_120490.1	206	67253	glucose-6-phosphate isomerase	no	yes	LdBPK_120010600	glucose-6-phosphate isomerase
LdBPK_050350.1	206	53530	trypanothione reductase	no	yes	LdBPK_050008500	trypanothione reductase
LdBPK_342440.1	195	53848	ATP-dependent DNA helicase, putative	no	yes	LdBPK_340031800	ruvB-like DNA helicase, putative
LdBPK_351420.1	194	90515	threonyl-tRNA synthetase, putative	no	yes	LdBPK_350019300	threonyl-tRNA synthetase, putative
LdBPK_367280.1	187	52781	protein disulfide isomerase	yes	no	LdBPK_360082200	protein disulfide isomerase 2
LdBPK_312210.1	184	31778	prostaglandin f2-alpha synthase/D-arabinose dehydrogenase	no	yes	LdBPK_310030900	prostaglandin f2-alpha synthase/D-arabinose dehydrogenase
LdBPK_332700.1	176	56614	aminopeptidase, putative	no	yes	LdBPK_330035200	metallo-peptidase, Clan MF, Family M17
LdBPK_091070.1	173	67608	hypothetical protein, conserved	yes	no	LdBPK_090016500	Present in the outer mitochondrial membrane proteome 10
LdBPK_190150.1	164	43016	aminopeptidase, putative	no	yes	LdBPK_190007200	metallo-peptidase, Clan MG, Family M24
LdBPK_353390.1	156	52423	6-phosphogluconate dehydrogenase, decarboxylating, putative	no	yes	LdBPK_350039100	6-phosphogluconate dehydrogenase, decarboxylating, putative
LdBPK_342410.1	151	22663	hypothetical protein, conserved	no	yes	LdBPK_340031500	Alba, putative

S1 Table continued from previous page

v1 ID	Score	Mass	v1 gene product	SignalP	Exosomes	v2 ID	v2 gene product
LdBPK_020340.1	149	59366	proteasome regulatory non-ATPase subunit 6, putative	no	yes	LdBPK_020009000	proteasome regulatory non-ATPase subunit 6, putative
LdBPK_352400.1	138	54431	aminopeptidase P, putative	no	yes	LdBPK_350029100	aminopeptidase P, putative
LdBPK_250780.1	130	45740	protein phosphatase, putative	no	yes	LdBPK_250013200	protein phosphatase, putative
LdBPK_321910.1	127	21742	iron superoxide dismutase, putative	no	yes	LdBPK_320024000	iron superoxide dismutase, putative
LdBPK_250940.1	113	19147	cyclophilin a	no	yes	LdBPK_250014800	cyclophilin type peptidyl-prolyl cis-trans isomerase
LdBPK_111160.1	113	83433	eukaryotic release factor 3, putative	no	yes	LdBPK_110017600	eukaryotic release factor 3, putative
LdBPK_251790.1	109	38482	pyruvate dehydrogenase E1 beta subunit, putative	yes	no	LdBPK_250023600	pyruvate dehydrogenase E1 beta subunit, putative
LdBPK_282970.1	108	34864	activated protein kinase c receptor (LACK)	no	yes	LdBPK_280034700	receptor for activated C kinase 1
LdBPK_323110.1	103	16728	nucleoside diphosphate kinase b	no	yes	LdBPK_320036600	nucleoside diphosphate kinase b
LdBPK_030500.1	612	99373	hypothetical protein, conserved	no	yes	LdBPK_030010000	RNA recognition motif. (a.k.a. RRM, RBD, or RNP domain), putative
LdBPK_141540.1	115	82868	hypothetical protein, conserved	no	no	LdBPK_140021000	C2 domain containing protein, putative
LdBPK_311930.1	110	14958	ubiquitin-fusion protein	no	no	LdBPK_360044200	polyubiquitin, putative
LdBPK_332400.1	702	33423	hypothetical protein, conserved	no	no	LdBPK_330032100	hypothetical protein, conserved
