# Supplementary material for: Refining wet lab experiments with in silico searches: A rational quest for diagnostic peptides in visceral leishmaniasis
Source: PLoS Negl Trop Dis. 2019 May 6;13(5):e0007353. doi: 10.1371/journal.pntd.0007353 (PMC6522066; doi:10.1371/journal.pntd.0007353)
Supplement: S2 Table — The top 20 scoring peptides from each of the four prediction algorithms were shortlisted and synthesised for antigenicity screening with Sudanese VL serum samples. ‘C.reported’ was calculated by dividing the reported amount by the volume of the solvent mixture in which the peptides were dissolved, while ‘C.280’ and ‘C.205’ were calculated based on the molar absorbances at 280 and 205 nm, respectively. All concentrations are given in mg/ml. MW is given in g/mol. Peptides that could not be dissolved and were therefore not included in the pilot peptide screening are marked with an asterisk. (PDF) [file pntd.0007353.s003.pdf]

S2 Table. The top 20 scoring peptides from each of the four prediction algorithms were shortlisted and synthesised for antigenicity screening with Sudanese visceral leishmaniasis (VL) serum samples. 'C.reported' was calculated by dividing the reported amount by the volume of the solvent mixture in which the peptides were dissolved, while 'C.280' and 'C.205' were calculated based on the molar absorbances at 280 and 205 nm, respectively. All concentrations are given in mg/ml. MW is given in g/mol. Peptides that could not be dissolved and were therefore not included in the pilot peptide screening are marked with an asterisk.

**ID**	**Sequence**	**Peptide length**	**MW**	**Purity**	**C.reported**	**C.280**	**C.205**
abc_01_CVTQ	CVTQEHFREAMAKTNP	16	2287.63	0.58	2	NA	3.27
abc_02_KEAP	KEAPGATEKDRAKATP	16	2095.38	0.57	2	NA	3.14
abc_03_TAYI	TAYIMRPLDHGADVTL	16	2198.57	0.69	2	2.75	NA
abc_04_MITN	MITNDDAPVRDSVLTD	16	2187.45	0.45	1.23	NA	NA
abc_05_MPTV*	MPTVDERQTFMFSATF	16	2333.71	0.4	2.31	NA	NA
abc_06_TRTG*	TRTGDYAFSYDKMLDM	16	2339.67	0.55	1.13	NA	NA
abc_07_KSTI	KSTISGHLLMEKGLVD	16	2153.57	0.53	2	NA	2.65
abc_08_KEAI	KEAITTFREEDPKVTD	16	2304.58	0.48	2	NA	NA
abc_09_HVYS	HVYSELGKKFGAAADP	16	2115.41	0.51	2	3.42	4.23
abc_10_LGMG	LGMGISGGEEGARKGP	16	1941.23	0.56	2	NA	2.52
abc_11_VAYQ	VAYQETPESERAELPP	16	2241.48	0.52	2	2.22	2.13
abc_12_AGTG	AGTGFPYREMMPMNAP	16	2195.61	0.72	2	3.95	NA
abc_13_TDSW*	TDSWGFFGVFDGHVND	16	2225.4	0.57	1.33	NA	NA
abc_14_HKKS	HKKSTEDNDDDAFCAP	16	2218.37	0.54	1	NA	NA
abc_15_YGLA	YGLAFDPYGGTAGLYD	16	2105.33	0.54	1.33	0.74	0.76
abc_16_WEEW	WEEWGNPNEYKYYDYM	16	2612.86	0.49	1.73	1.68	1.43
abc_17_HSTI	HSTIGVHPTSAEELCS	16	2093.33	0.65	2	NA	2.68
abc_18_EKCI	EKCIELKPDFVKGYAR	16	2321.76	0.53	0.87	3.85	1.08
abc_19_PAGI	PAGINIPNYDDIRQTV	16	2211.5	0.57	0.77	1.69	0
abc_20_TGTI	TGTIDNGVVKMEKAEE	16	2146.44	0.55	2	NA	NA
BP_01_CSPP	CSPPPPSPSPHPRPPS	16	2062.38	0.7	2	NA	1.98
BP_02_GEGS	GEGSPTSPTSPKQPGS	16	1939.11	0.65	2	NA	2.08
BP_03_AEAG	AEAGAPAGSGAPPPAD	16	1760.92	0.56	2	NA	NA

S2 Table continued from previous page

ID	Sequence	Peptide length	MW	Purity	C.reported	C.280	C.205
BP_04_KPAP	KPAPPKPKESKEPENA	16	2172.55	0.59	2	NA	1.88
BP_05_TAKP	TAKPKQQDEDPDGAAE	16	2125.28	0.63	2	NA	NA
BP_06_GGDR	GGDRGGGTGNEDDDYE	16	2039.02	0.79	2	2.95	2.05
BP_07_MQQP	MQQPPTPQPQKQQKQQ	16	2345.71	0.53	2	NA	3.13
BP_08_EEEE*	EEEEEEEEEEEPQATR	16	2417.44	0.61	1.62	NA	NA
BP_09_RSDP	RSDPSGGGGGNRDDNE	16	2015.04	0.6	2	NA	NA
BP_10_APAS	APASAPAPAPAAAPTP	16	1783.06	0.67	2	NA	2.61
BP_11_KRGG	KRGGDKDGGGESGEAA	16	1916.04	0.62	2	NA	NA
BP_12_RSQQ	RSQQGEQEPEEDEEEV	16	2343.41	0.63	2	NA	NA
BP_13_QQLS	QQLSSSPPPPRERAED	16	2219.45	0.57	2	NA	NA
BP_14_EPEV	EPEVGEEPQPEEEEDA	16	2238.31	0.72	2	NA	NA
BP_15_AAPS	AAPSGGPSGNSDEEDL	16	1928	0.68	2	NA	NA
BP_16_SDVT	SDVTGGGGGGGSGGGG	16	1560.6	0.64	1	NA	NA
BP_17_GQQQ	GQQQQQQDPPAGQQGV	16	2119.28	0.82	2	NA	NA
BP_18_AETP	AETPADDAGQPHEPEK	16	2117.26	0.71	2	NA	NA
BP_19_PPSA	PPSAGSKDGAPSDGVP	16	1864.05	0.51	2	NA	NA
BP_20_DATP	DATPAAANGEGPGKEN	16	1924.06	0.65	2	NA	NA
EpQ_01_AYAT	AYATMLKDVQWKVRKS	16	2349.82	0.58	2	6.79	6.25
EpQ_02_HEKL	HEKLVQDIWKKLEAKG	16	2347.79	0.58	2	4.28	4.01
EpQ_03_SCSV	SCSVKLGLWKNAVNNC	16	2161.55	0.57	2	6.56	6.9
EpQ_04_MAYV	MAYVCELGPNQGWK	14	2021.38	0.58	0.88	6.49	NA
EpQ_05_LKDP	LKDPKQYQSIVDAEWK	16	2373.73	0.31	2	1.88	NA
EpQ_06_ERCE	ERCEDPNAWKGPTNGG	16	2156.35	0.62	1.33	1.92	NA
EpQ_07_DNPA	DNPAGPTTWKSDEPAL	16	2124.33	0.62	1.73	1.8	1.88
EpQ_08_YGIS	YGISFPKNPMLTEWKT	16	2337.77	0.43	2	2.69	2.79
EpQ_09_GMSS	GMSSDQLLQFLLQQQQ	16	2289.63	0.48	1.86	NA	NA
EpQ_10_AAKK	AAKKKRVGCWK	11	1700.11	0.56	2	2.29	2.22

S2 Table continued from previous page

ID	Sequence	Peptide length	MW	Purity	C.reported	C.280	C.205
EpQ_11_NIRI	NIRIHLGDTIRIAPCK	16	2245.71	0.51	1.1	NA	2.33
EpQ_12_ERRR	ERRRVEYQQFLDVCGQ	16	2451.78	0.41	1.4	6.14	NA
EpQ_13_MDRE	MDRESLCPNWK	11	1804.11	0.41	2	2.06	NA
EpQ_14_SRQM	SRQMTMCKEERIANCK	16	2353.81	0.34	2	NA	1.85
EpQ_15_DWSI	DWSIVERGWK	10	1700.95	0.5	2.13	1.69	NA
EpQ_16_VLVQ*	VLVQGAIWGINSYDQW	16	2274.6	0.39	15	NA	NA
EpQ_17_GRTI	GRTILRNHKWAGNNKV	16	2289.67	0.31	2	2.1	1.79
EpQ_18_EKVR	EKVRSGEWKGQTGKSI	16	2215.53	0.38	2	2.13	2.11
EpQ_19_PDVA	PDVAHVVQFDLPQEMD	16	2265.57	0.52	2.31	NA	NA
EpQ_20_ADVT	ADVTATLAWK	10	1500.75	0.49	1.03	1.56	NA
LbT_01_RPGG	RPGGPPGYRTPYTAK	15	2043.35	0.45	2	9.28	2.46
LbT_02_TQGD	TQGDRQKIQDAVSAA	15	2013.23	0.52	1.3	NA	NA
LbT_03_EVKS	EVKSRYNVDVSQNKR	15	2247.54	0.53	2	2.69	2.1
LbT_04_VIEM	VIEMTRAFEDDDFDK	15	2256.51	0.51	2	NA	NA
LbT_05_GSAD	GSADLTPSNLTRPAS	15	1912.12	0.47	2	NA	NA
LbT_06_VRPI	VRPIPSFDDMPLHQN	15	2191.53	0.47	2	NA	NA
LbT_07_CELG	CELGPNQGWKAVVAD	15	2012.3	0.47	1.07	1.59	NA
LbT_08_APQQ	APQQTQSGIRRVTRA	15	2094.4	0.44	2	NA	2.18
LbT_09_SAEE	SAEEKGTGKRNQITI	15	2057.33	0.42	2	NA	0.7
LbT_10_GEAE	GEAEWLEWESTVLTP	15	2172.42	0.54	0.96	1.08	0
LbT_11_EELR	EELRQRRHQGPGSPG	15	2129.36	0.41	0.87	NA	0.66
LbT_12_EWAN	EWANKPLDDLDPHPS	15	2159.38	0.55	2	1.57	1.56
LbT_13_IKEE*	IKEETEMIEGEVVEV	15	2159.48	0.5	1.62	NA	NA
LbT_14_VSDF	VSDFFGGKELNKSIN	15	2080.36	0.74	2.92	NA	2.12
LbT_15_WEDV	WEDVGGLLDVKRELQ	15	2182.5	0.53	2	1.67	0
LbT_16_SGGA	SGGAGPAGGASSGPK	15	1582.72	0.53	2	NA	2.5
LbT_17_KATN	KATNGDTHLGGEDFD	15	2002.12	0.57	2	NA	NA

S2 Table continued from previous page

ID	Sequence	Peptide length	MW	Purity	C.reported	C.280	C.205
LbT_18_EREG	EREGKDITLIGFSRG	15	2103.4	0.53	1.56	NA	2
LbT_19_AWAS	AWASSPAPTEARTAP	15	1938.17	0.45	2	4.01	NA
LbT_20_VDRD	VDRDNKKLSSGMVCS	15	2064.4	0.51	2	NA	2.53
